# SPI-Hub™: a gateway to scholarly publishing information

**DOI:** 10.5195/jmla.2020.815

**Published:** 2020-04-01

**Authors:** Taneya Y. Koonce, Mallory N. Blasingame, Jerry Zhao, Annette M. Williams, Jing Su, Spencer J. DesAutels, Dario A. Giuse, John D. Clark, Zachary E. Fox, Nunzia Bettinsoli Giuse

**Affiliations:** Associate Director for Research, Center for Knowledge Management, Strategy and Innovation, Vanderbilt University Medical Center, Nashville, TN, taneya.koonce@vumc.org, https://orcid.org/0000-0002-4014-467X; Information Scientist, Center for Knowledge Management, Strategy and Innovation Vanderbilt University Medical Center, Nashville, TN, mallory.n.blasingame@vumc.org, https://orcid.org/0000-0003-0356-9481; Senior Application Developer, Center for Knowledge Management, Strategy and Innovation, Vanderbilt University Medical Center, Nashville, TN, jerry.zhao@vumc.org; Senior Information Scientist, Center for Knowledge Management, Strategy and Innovation, Vanderbilt University Medical Center, Nashville, TN, annette.williams@vumc.org, https://orcid.org/0000-0002-2526-3857; Information Scientist, Center for Knowledge Management, Strategy and Innovation, Vanderbilt University Medical Center, Nashville, TN, jing.su@vumc.org; Information Scientist, Center for Knowledge Management, Strategy and Innovation, Vanderbilt University Medical Center, Nashville, TN, spencer.desautels@vumc.org, https://orcid.org/0000-0002-6120-2496; Associate Professor, Department of Biomedical Informatics, Vanderbilt University School of Medicine and Vanderbilt University Medical Center, Nashville, TN, dario.giuse@vumc.org; Senior Application Developer, Center for Knowledge Management, Strategy and Innovation, Vanderbilt University Medical Center, Nashville, TN, john.clark@vumc.org; Associate Director for Information Services, Center for Knowledge Management, Strategy and Innovation, Vanderbilt University Medical Center, Nashville, TN, zachary.e.fox@vumc.org; Professor of Biomedical Informatics and Professor of Medicine; Vice President for Knowledge Management; and Director, Center for Knowledge Management, Strategy and Innovation, Vanderbilt University Medical Center, Nashville, TN, nunzia.giuse@vanderbilt.edu, https://orcid.org/0000-0002-7644-9803

## Abstract

**Background:**

Advances in the health sciences rely on sharing research and data through publication. As information professionals are often asked to contribute their knowledge to assist clinicians and researchers in selecting journals for publication, the authors recognized an opportunity to build a decision support tool, SPI-Hub: Scholarly Publishing Information Hub™, to capture the team’s collective publishing industry knowledge, while carefully retaining the quality of service.

**Case Presentation:**

SPI-Hub’s decision support functionality relies on a data framework that describes journal publication policies and practices through a newly designed metadata structure, the Knowledge Management Journal Record™. Metadata fields are populated through a semi-automated process that uses custom programming to access content from multiple sources. Each record includes 25 metadata fields representing best publishing practices. Currently, the database includes more than 24,000 health sciences journal records. To correctly capture the resources needed for both completion and future maintenance of the project, the team conducted an internal study to assess time requirements for completing records through different stages of automation.

**Conclusions:**

The journal decision support tool, SPI-Hub, provides an opportunity to assess publication practices by compiling data from a variety of sources in a single location. Automated and semi-automated approaches have effectively reduced the time needed for data collection. Through a comprehensive knowledge management framework and the incorporation of multiple quality points specific to each journal, SPI-Hub provides prospective users with both recommendations for publication and holistic assessment of the trustworthiness of journals in which to publish research and acquire trusted knowledge.

## BACKGROUND

Journal trustworthiness and rigor have been much discussed since the transition to electronic publishing in the 1990s [1, 2]. The broadening of access to the literature, concomitant with the ability to read full-text articles online and on demand, brought an impetus to remove additional barriers posed by licensing and permissions restrictions through open access [3]. A recent analysis has found that open access publications constitute “at least 28% of the scholarly literature” across all disciplines [4], and many sponsors now require that authors make grant-supported findings openly available as a condition of funding to ensure barrier-free dissemination of research [5–10]. While the benefits of open access are significant [4, 11, 12], the opening of the academic publishing market has also led to unintended consequences.

The shift in the payment model, whereby funding often derives from author-paid article processing charges, has created an opportunity for players who are less driven by standards to earn a profit potentially using misleading or non-transparent practices [13, 14]. The exploitation of the pay-for-publication model threatens the integrity of the scientific communication process and confounds the ability to assess journal quality [15–17]. A 2019 study reported that more than 150 systematic reviews and meta-analyses used content from a biomedical publisher against whom the Federal Trade Commission took legal action for deceptive business practices [18]. It is, thus, impossible to tell if ethical standards were maintained or peer review was performed. Researchers must be aware of journal assessment methods both to avoid using literature that is of unknown, possibly low, quality, as well as to avoid the risk of sending high-quality research to low-quality journals, which may result in reputational harm, low discoverability, and potential disappearance from the academic record [19, 20].

Long-established bibliographic databases—such as Ulrichsweb, OCLC’s WorldCat, and the National Library of Medicine (NLM) Catalog [21–23]—offer objective, fact-based descriptive journal metadata. Such databases represent a journal at a single point in time, but unequivocal, current descriptive information requires consulting journal and/or publisher websites directly.

To assist authors in making decisions about where to publish, several organizations have issued guidance and checklists, including: Think. Check. Submit. [24], the Directory of Open Access Journals [25], and the World Association of Medical Editors algorithm [26]. Consistent among these assessment tools is an emphasis on considering multiple journal characteristics to assess a publication’s approach to editorial practices and commitment to transparency. Weighing multiple journal characteristics is key, as “one-stop” approaches to identifying whether a journal is “legitimate” have proved elusive and controversial [27–29], and reliance on a single element such as the journal’s inclusion in a specific database (citation or full-text) can be misleading [30].

However, finding each component of information for multiple journals can be a time-consuming and daunting process. Several tools attempt to automate journal selection for potential authors and facilitate quick discovery of relevant journals for publication, based on scope and other elements such as impact metrics [31–41]. In the authors’ experience, such tools often have one or more shortcoming: lack of details that specifically indicate a journal’s publication rigor and transparency as defined by the previously mentioned guidelines, journal inclusion limited to a specific publisher, commercial affiliations with companies specializing in manuscript preparation and editing services, availability via subscription services only, and subjective assessments of journal quality.

There is a clear challenge in evaluating quality and validity in the increasingly complex world of publishing. As has been our experience at the Center for Knowledge Management at Vanderbilt University Medical Center, information specialists are often called upon to assist clinicians and researchers in identifying where to publish their research and, in the process, steer them away from journals that lack rigor. As requests for assistance with journal investigation and selection have become more frequent, our team has recognized the need to scale the process through an innovative approach to automating its information specialists’ collective knowledge of the publishing industry. Making the process scalable allows us to provide this important service while freeing our team’s limited resources to address other challenges. Knowledge management approaches—such as knowledge curation, data organization, and content maintenance—can be directly applied to help guide clinicians and researchers through this dynamic landscape.

Our team’s experience applying knowledge management strategies to inform clinical decision support [42, 43] was foundational as we developed our decision support tool for identifying journals and evaluating their transparency and rigor: the SPI-Hub: Scholarly Publishing Information Hub™. The tool’s features and supporting metadata infrastructure, which we have coined the “Knowledge Management Journal Record™” [44], has facilitated the appraisal of journal transparency for informing authors’ decision-making about publication venues as well as for clinicians and researchers who need to critically evaluate journals [45].

## CASE PRESENTATION

### Knowledge Management Journal Record™

Our center’s approach to identifying where to publish manuscripts has traditionally involved manually capturing multiple data points to generate a snapshot of a journal’s scope and publishing policies. We have leveraged our manual process, combined with best practices from the aforementioned criteria and checklists, to define the metadata for the Knowledge Management Journal Record. The record focuses on multiple journal rigor and quality data points that can support informed decision-making for all journal types and avoids prescribing subjective assessments. The Knowledge Management Journal Record is intentionally designed to present users with impartial, current data about journals in as objective a manner as possible, allowing users to judge which elements of the journal record are most important to them.

The current fields in the Knowledge Management Journal Record have been selected based on a comprehensive review of publication guidelines and standards and the ease with which fields could be captured in a structured and/or semi-automated manner. The fields are organized in four sections: “General Information” (e.g., publication frequency, publication start year); “Metrics & Indexing” (e.g., MEDLINE indexing status); “Publication Policies” (e.g., Committee on Publication Ethics membership status, archiving status); and “Open Access” (e.g., Directory of Open Access Journals inclusion status, Creative Commons licenses offered). Supplemental Appendix A provides a detailed description, rationale, and data source for each of the twenty-five fields.

### SPI-Hub: Scholarly Publishing Information Hub™ features

Leveraging the Knowledge Management Journal Record as its core infrastructure, we created SPI-Hub as a decision support tool for journal identification and assessment. SPI-Hub currently includes three primary functionalities: “Search by Topic,” “Search by Journal,” and “Search by Author.” Links to journal suggestion and selection resources that are publicly available are also provided in a “Resources” tab for prospective users to consult as a complement to SPI-Hub’s information. A “Contact Us” page allows users to provide feedback and suggest journals to add to the SPI-Hub database. The “Contact Us” page has a tab with Frequently Asked Questions (supplemental Appendix B), which provides answers to questions received from users’ feedback that we are working to address in the next version of SPI-Hub. This page will be updated on an ongoing basis as suggestions are implemented and we continue to expand and refine SPI-Hub.

#### Search by Topic

To help prospective authors find journals that publish works on a given topic, the tool offers a multifaceted search against PubMed. Using the Entrez Programming Utilities interface (E-utilities) provided by the National Center for Biotechnology Information (NCBI), we first recommend subject keywords from the PubMed autocompleter [46] for user search terms; free text keywords are also allowed. The NCBI E-utilities interface is used to retrieve PubMed citations and abstracts inclusive of all matching terms; search results are then aggregated at the journal level; and a ranked list of active journals publishing on the user’s submitted topic is generated.

**Figure 1:**
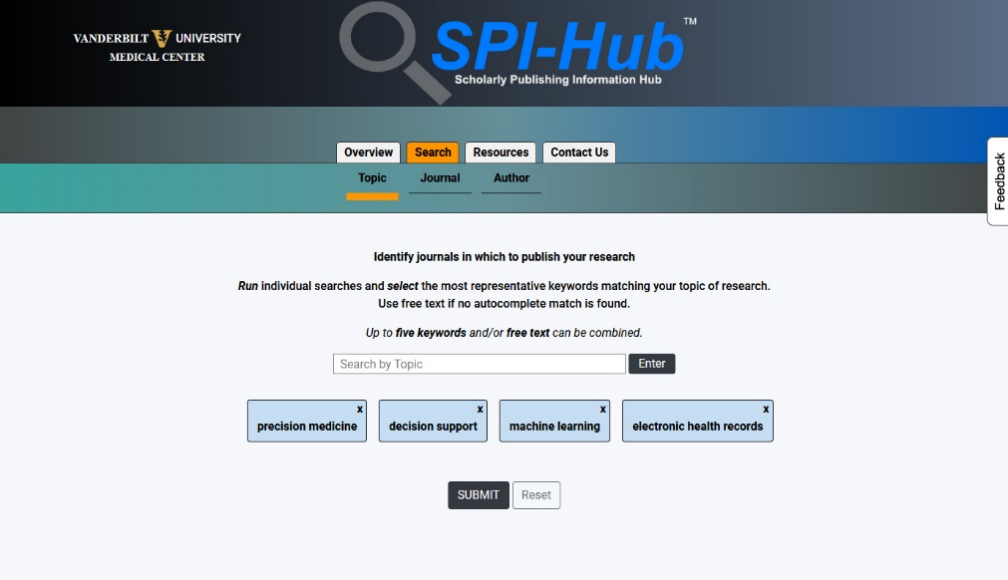
Example Search by Topic

**Figure 2:**
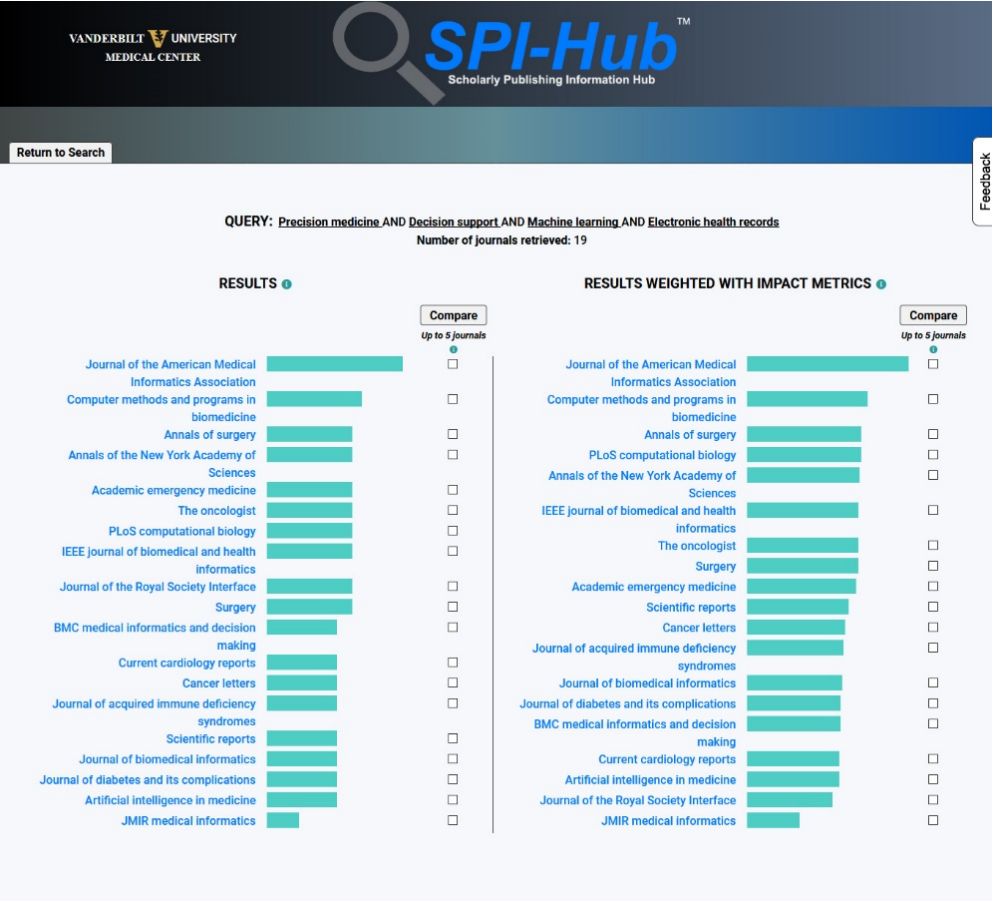
Journal results from Search by Topic option

**Figure 3:**
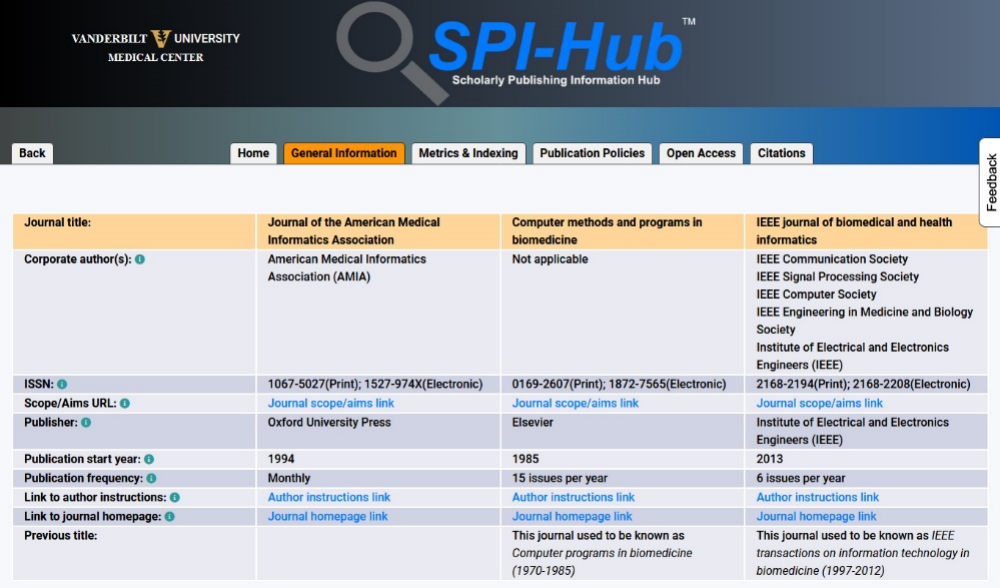
Example journal comparison screen

The ranking uses a weighted algorithm that is largely informed by the team’s expertise and knowledge of authorship rules and regulations, publication standards, and the publishing industry. Results are displayed in two columns, with one column showing the default results ranking and a second column with citation-based impact metrics added to the weighting, because impact metrics are often used by authors as a criterion for journal selection but have known limitations as indicators of journal quality [47–49]. Once results are returned, users can view the record of each journal or, alternatively, select up to three journals for a comparison view. Information buttons are included throughout the record to provide brief field definitions to aid users in assessing the information in any specific metadata field. Figures 1, 2, and 3 show an actual search for an informatics faculty member, recently conducted using SPI-Hub, to identify journals to submit a research article in the areas of “precision medicine,” “decision support,” “machine learning,” and “electronic health records.”

#### Search by Journal Name

The journal name search allows users to find detailed information about a specific journal. This function searches across all names and alternate titles of a journal in our database and utilizes an autocompleter for convenience. Upon a successful match, SPI-Hub retrieves the Knowledge Management Journal Record entry for the journal (supplemental Appendix C).

#### Search by Author

The author search functionality provides another mechanism for identifying potential publication venues by allowing the user to quickly view the scholarly journals in which colleagues working in similar areas of interest and research have published. The interface allows the user to input structured citation lists that may constitute an online curriculum vitae, including an ORCID identifier, an NCBI My Bibliography link, or the public uniform resource locator (URL) of a Zotero personal library or group [50–52]. This selection was informed both by prevalence of use [53–55] and by the provision of citation information via structured formats that can be parsed for comparisons to journals in SPI-Hub. Once journal matches are identified, the tool returns a list of hyperlinks to SPI-Hub records (supplemental Appendix D).

### Data sources, automation, and verification

To initially populate the database, we downloaded the “List of All Journals Cited in PubMed” [56] and used the NCBI E-utilities to access the NLM Catalog to extract multiple data elements for each Knowledge Management Journal Record. For more comprehensive coverage, additional biomedical journals that were not indexed in PubMed were added, based on our team’s knowledge of publication venues, via email solicitations, online news articles, and social media postings, with emphasis on the publishers with the largest number of journals [57]. We supplemented the information downloaded from NLM by extracting data from multiple external sources via an application programming interface (API) and matching the data to SPI-Hub records (e.g., by comparing International Standard Serial Number [ISSN]) so that fields could be populated accordingly.

Based on patterns observed through manual completion of over 2,000 records, some fields are populated using information from publisher websites through semi-automated methods and manual review. When possible, publisher-level policies are established that apply to multiple journals. For example, a publisher may state that all of their journals undergo double-blind peer review, which enables us to create a standardized message for every journal produced by that publisher. Automated data accuracy and integrity checks are run periodically and used to update the database as new information becomes available from data sources.

### Status of implementation

SPI-Hub includes approximately 24,000 currently active journal titles. Record completion is ongoing, with priority given to the journals in which our institution’s authors have most commonly published as well as the journals with the largest publishers, because those journals allow the record to be rapidly completed through automation of publisher-specific data. An internal study conducted to assess resource allocation for project completion and future maintenance, which detailed 4 distinct stages of automation, showed that the team was able each time to reduce the average time needed to fully complete journal records (supplemental Appendix E).

The last stage of automation, which is applicable to journals from large publishers, requires manual intervention only for 3 of the 25 metadata fields, showing a time reduction from 12.16 to 1.62 minutes to complete an entire record. With the introduction of automation, variances and errors were also greatly reduced, resulting in less time needed for quality control during the data acquisition phase of the project. To date, more than 16,100 records are fully completed in our database, with the remainder at least 50% complete.

## DISCUSSION

SPI-Hub is a decision support application to aid researchers in identifying journals in which to publish and for reviewing journal transparency and rigor. A user can quickly review all information in a Knowledge Management Journal Record holistically to gain important insights into a single journal. By comparing two or more records, a user can identify and evaluate differences. For example, the integration into the record of data from different sources allows users to distinguish between a journal’s self-reported impact metrics and a verifiable JCR impact factor [58–60] and identify any discrepancies, while also reporting, when applicable, the existence of other impact metrics such as SciMago journal rank or CiteScore. Other fields such as peer review and open access policies rely on a journal’s self-reported practices. While the tool accurately reflects the journal’s stated peer-review policy, other methods may be required to fully assess the quality of peer review [61]. Results of test searches (Figures 1–3) very closely represent the types of results and feedback that the team provides to users through a manual process, thus demonstrating the potential value SPI-Hub has in assisting information professionals.

Our automation study demonstrates the efficiency of the automated and semi-automated techniques applied to SPI-Hub. Importantly, it shows that in our current work flow, the time needed to complete Knowledge Management Journal Records can be considerably shorter than with a manual process. These automation findings have important implications, as the significant reduction of the manual effort improves the feasibility of keeping the information current. This will be key to the long-term sustainability of SPI-Hub.

As we further develop SPI-Hub, a robust maintenance strategy is also being implemented and will be key to the tool’s ongoing success and usefulness. This strategy includes periodic assessment of changes and updates to journal websites and third-party data sources alike. Maintenance, with all of its challenges, requires an ongoing, significant behind-the-scenes effort because journal details (e.g., publisher, publication frequency, indexing status in biomedical databases) change over time and sources change their data structure. For example, the Committee on Publication Ethics’s most recent change on how to search its membership data has already necessitated an update of our automated data gathering.

While SPI-Hub offers clear benefit as a decision support aid for journal identification and selection, there are two important limitations to report. First, while much progress has been made in the automation of data collection, more work is needed to fully automate the process. Second, at this stage, no effort has been made by the team to collect feedback from papers’ authors and peer reviewers about their experience working with specific journals or publishers, which could provide additional information about whether the journal or publisher actually adheres to its stated policies.

Our approach offers the benefit of an open and transparent process: SPI-Hub makes every effort to provide unbiased, factual information by which users can perform journal quality assessments. All stakeholders recognize how critical it is that researchers and individuals who are seeking journals in which to publish are able to assess their rigor and transparency. Through a comprehensive knowledge management framework and the incorporation of multiple quality points specific to each journal, SPI-Hub provides an opportunity for holistic assessment of the trustworthiness of journals in which to publish research and acquire trusted knowledge.

Through a series of planned rollouts, beta testing, and collection of anonymous user feedback (supplemental Appendix F), SPI-Hub has been under a process of constant improvement and refinement. Planned changes to the system, informed by this process, are being communicated to our users on an ongoing basis through a regularly updated Frequently Asked Questions page (supplemental Appendix B). At the conclusion of this phase of rapid refinement, SPI-Hub will be released to the general public and undergo a formal evaluation.

## SUPPLEMENTAL FILES

Appendix A Knowledge Management Journal Record™ rationale and data sources

Appendix B SPI-Hub™ Frequently Asked Questions (FAQ) feature

Appendix C Sample Knowledge Management Journal Record™

Appendix D Search by Author functionality

Appendix E Automation impact study

Appendix F SPI-Hub™ user evaluation

## 

**Taneya Y. Koonce, MSLS, MPH**, taneya.koonce@vumc.org, https://orcid.org/0000-0002-4014-467X, Associate Director for Research, Center for Knowledge Management, Strategy and Innovation, Vanderbilt University Medical Center, Nashville, TN

**Mallory N. Blasingame, MA, MSIS**, mallory.n.blasingame@vumc.org, https://orcid.org/0000-0003-0356-9481, Information Scientist, Center for Knowledge Management, Strategy and Innovation Vanderbilt University Medical Center, Nashville, TN

**Jerry Zhao, MS, MLIS**, jerry.zhao@vumc.org, Senior Application Developer, Center for Knowledge Management, Strategy and Innovation, Vanderbilt University Medical Center, Nashville, TN

**Annette M. Williams, MLS**, annette.williams@vumc.org, https://orcid.org/0000-0002-2526-3857, Senior Information Scientist, Center for Knowledge Management, Strategy and Innovation, Vanderbilt University Medical Center, Nashville, TN

**Jing Su, MD, MS**, jing.su@vumc.org, Information Scientist, Center for Knowledge Management, Strategy and Innovation, Vanderbilt University Medical Center, Nashville, TN

**Spencer J. DesAutels, MLIS**, spencer.desautels@vumc.org, https://orcid.org/0000-0002-6120-2496, Information Scientist, Center for Knowledge Management, Strategy and Innovation, Vanderbilt University Medical Center, Nashville, TN

**Dario A. Giuse, Dr. Ing., MS, FACMI**, dario.giuse@vumc.org, Associate Professor, Department of Biomedical Informatics, Vanderbilt University School of Medicine and Vanderbilt University Medical Center, Nashville, TN

**John D. Clark, MS**, john.clark@vumc.org, Senior Application Developer, Center for Knowledge Management, Strategy and Innovation, Vanderbilt University Medical Center, Nashville, TN

**Zachary E. Fox, MSIS**, zachary.e.fox@vumc.org, Associate Director for Information Services, Center for Knowledge Management, Strategy and Innovation, Vanderbilt University Medical Center, Nashville, TN

**Nunzia Bettinsoli Giuse, MD, MLS, FACMI, FMLA**, nunzia.giuse@vanderbilt.edu, https://orcid.org/0000-0002-7644-9803, Professor of Biomedical Informatics and Professor of Medicine; Vice President for Knowledge Management; and Director, Center for Knowledge Management, Strategy and Innovation, Vanderbilt University Medical Center, Nashville, TN
