# Supplementary material for: SPI-Hub™: a gateway to scholarly publishing information
Source: J Med Libr Assoc. 2020 Apr 1;108(2):286–94. doi: 10.5195/jmla.2020.815 (PMC7069808; doi:10.5195/jmla.2020.815)
Supplement: Appendix A [file jmla-108-286-s001.pdf]

JMLA Journal of the Medical Library Association

Supplemental content to
J Med Libr Assoc. Apr;108(2):dx.doi.org/10.5195/jmla.2020.815
www.jmla.mlanet.org
© Koonce, Blasingame, Zhao, Williams, Su, DesAutels, Giuse, Clark, Fox, Giuse 2020

SPI-Hub™: a gateway to scholarly publishing information

Taneya Y. Koonce, MSLS, MPH; Mallory N. Blasingame, MA, MSIS; Jerry Zhao, MS, MLIS; Annette M. Williams, MLS; Jing Su, MD, MS; Spencer J. DesAutels, MLIS; Dario A. Giuse, Dr.Ing., MS, FACMI; John D. Clark, MS; Zachary E. Fox, MSIS; Nunzia Bettinsoli Giuse, MD, MLS, FACMI, FMLA

APPENDIX A

Knowledge Management Journal Record™ rationale and data sources

Knowledge Management Journal Record™ fields were defined after review of the literature for (1) guidelines, checklists, and guidance documents that promote sound publication practices; (2) published analyses of characteristics associated with journal quality; (3) criteria established by existing tools that aid in journal selection; and (4) standards for cataloging journal records. Based on these findings, the authors compiled a list of fields and reached consensus on data elements that are necessary to include in the record. Each Knowledge Management Journal Record is organized into four sections: (1) General Information about the journal, (2) Metrics & Indexing practices, (3) Publication Policies, and (4) Open Access details. The twenty-five data points provided in the record, in total, aid the understanding of a journal's rigor and transparency practices.

General Information

The details in this section of the record are useful for assessing if the journal title is reflective of its stated aims, implies an overly broad scope, or combines unrelated subjects; these attributes have all been identified as potential indicators of a lack of editorial focus or transparency [1–3]. Affiliation details provide context for readily recognizing a relationship with professional or trade organizations [4]. International Standard Serial Numbers (ISSNs) are provided, as their absence may be a potential warning sign for journals that do not adhere to best publishing practice [5]. Publication frequency details can be used to assess if a journal offers a clear and predictable publication schedule [1, 2, 6]. An easily identified publication start year gives further insight, as journals with longer publication histories may have established transparent practices and predate the exploitation of open access publishing models [7].

Metrics & Indexing practices

A plethora of metrics exists to ascertain the publishing impact of a journal (or its included articles). While some impact metrics, such as the Clarivate's Journal Citation Reports (JCR) impact factor, are only assigned to journals that meet specific quality criteria, other journal metrics might not evaluate journals for quality or rigor. Indicating in each record the specific impact metrics that a journal claims on its website aids the user in assessing the metrics with which each journal affiliates. Furthermore, if the journal claims to have an "impact factor," the record's inclusion of details from JCR provides a means to verify if that claim is true [3, 5, 7].

The journal's inclusion in well-known, highly curated databases such as MEDLINE and Science Citation Index Expanded (SCIE) also denotes that the journal has met specific quality criteria [1, 3–6]. Some journals have limited, select article inclusion in PubMed given the requirements of the National Institutes of Health (NIH) public access policy [8]. While the reason for this limited inclusion varies (e.g., journal is overall out of scope, journal is only selectively indexed), this information is provided in each Knowledge Management Journal Record, if applicable, because some less rigorous journals claim "Indexed in PubMed" status on their websites even when the only articles present in the PubMed database are NIH-funded articles that require PubMed Central deposit [9].

SC
Supplemental content to
J Med Libr Assoc. Apr;108(2):dx.doi.org/10.5195/jmla.2020.815
www.jmla.mlanet.org
© Koonce, Blasingame, Zhao, Williams, Su, DesAutels, Giuse, Clark, Fox, Giuse 2020

Publication Polices

The cornerstone of evaluating journal rigor and transparency is understanding publication policies and practices. For example, multiple resources [1–4, 6, 10] state that journals should clearly and overtly describe their peer-review and editorial practices and adhere to best practice recommendations established by organizations such as the International Committee of Medical Journal Editors (ICMJE) [11] and the Committee on Publication Ethics (COPE) [12]. Beyond statement of adherence to best practice recommendations, journal membership in organizations like COPE is further testament to a commitment to ethical publication practices [1, 3]. To ensure the long-term preservation of the scientific record and firmly establish longevity of access, journals should also commit to stable archiving methods, whether through PubMed Central or other services such as CLOCKSS [1–4, 6, 10, 13, 14].

Open Access

Given the exploitation of the open access model by publishers who engage in less than transparent and possibly unethical publication practices, the metadata fields in the Open Access section of each Knowledge Management Journal Record are especially insightful. Similar to COPE, the Open Access Scholarly Publishing Association (OASPA) specifically promotes ethical practices for open access journal publishers [6]. Membership in OASPA or a journal's inclusion in the Directory of Open Access Journals (DOAJ) can be an indicator of commitment to best practices in open access publishing [1]. While DOAJ provides a curated database of open access publications that adhere to a series of quality standards [10], journals that are awarded a DOAJ seal are recognized for their efforts to meet an "exceptionally high level of publishing standards" and the "highest level of 'openness'" [15]. Additional best practices for open access publications include clearly listing article processing charges (APCs) and offering licensing options (such as Creative Commons) that allow author retention of copyright and flexible sharing and reuse of article content [1, 3, 4, 10].

Tables 1 and 2 provide descriptions of data points in the metadata fields.

Supplemental Table 1 Knowledge Management Journal Record™ metadata field descriptions

Field name	Brief description
General Information	
Journal title	Title of the journal
Corporate author(s)	Professional societies or organizations affiliated with the journal
ISSN	The print and electronic ISSN numbers
Scope/aims uniform resource locator (URL)	Hyperlink to scope or aims statement on journal website
Publisher	Name of the journal publisher
Publication start year	The start year of the journal (or start year of the current title if journal has had a title change)
Publication frequency	Frequency of publication (e.g., weekly, monthly, bimonthly)
Link to author instructions	Hyperlink to author instructions page on journal website
Link to journal home page	Hyperlink to home page of journal website

JMLA Journal of the Medical Library Association

Supplemental content to
J Med Libr Assoc. Apr;108(2):dx.doi.org/10.5195/jmla.2020.815
www.jmla.mlanet.org
© Koonce, Blasingame, Zhao, Williams, Su, DesAutels, Giuse, Clark, Fox, Giuse 2020

Field name	Brief description
Metrics & Indexing	
Impact metrics listed on journal website	Article impact metrics reported on the journal website (e.g., JCR impact factor, Google Scholar h-5 index, Index Copernicus value, Scimago journal rank)
Verifiable JCR impact factor	Is the title included in the Clarivate JCR journals list?
MEDLINE indexing status	Statement to describe the journal's indexing status in NLM's MEDLINE database
Indexed in SCIE	Is the journal indexed in the database?
Publication Policies	
Statement of peer-review policy	Does the journal state a peer-review policy?
Adheres to ICMJE recommendations	Does the journal state adherence to ICMJE recommendations?
COPE member	Journal's membership status with COPE
Archived in PubMed Central	Journal's archiving status in PubMed Central
Participates in national and international archiving services (e.g., CLOCKSS)	Journal's participation in archiving services as documented in the Keepers Registry
Open Access	
Open access publication	Is the title an open access publication or is open access offered for authors on a per-article basis?
OASPA member	Is the publisher a member of OASPA?
Included in DOAJ	Is the journal included in the DOAJ directory?
DOAJ seal	If the journal is in DOAJ, has it been awarded a DOAJ Seal of Approval?
Clearly describes APCs	Does the journal clearly state the applicable article processing charges?
Statement of article copyright holder	Does the journal clearly state who holds copyright of an open access paper?
Creative Commons licenses offered	Are Creative Commons licenses offered and if so, which ones?

Abbreviations: APCs=article processing charges, COPE=Committee on Publication Ethics, DOAJ=Directory of Open Access Journals, ICMJE=International Committee of Medical Journal Editors, ISSN=International Standard Serial Number, JCR=Journal Citation Reports, NLM=National Library of Medicine, OASPA=Open Access Scholarly Publishing Association, SCIE=Science Citation Index Expanded.

JMLA Journal of the Medical Library Association

SC
Supplemental content to
J Med Libr Assoc. Apr;108(2):dx.doi.org/10.5195/jmla.2020.815
www.jmla.mlanet.org
© Koonce, Blasingame, Zhao, Williams, Su, DesAutels, Giuse, Clark, Fox, Giuse 2020

Supplemental Table 2 Knowledge Management Journal Record data sources, automation level, and stage of automation implementation

Field name	Primary source	Additional/confirmatory sources	Level of automation	Stage of automation implementation
General Information				
Journal title	NLM's List of all Journals Cited in PubMed	Not applicable	Automated	1
ISSN	NLM's List of all Journals Cited in PubMed	Journal/publisher website; ISSN portal	Semi-automated	3
Corporate authors	NLM Catalog	Journal/publisher website; society websites	Semi-automated	3*
Scope/aims URL	Journal website	Not applicable	Semi-automated	4
Publisher	NLM Catalog	Journal/publisher website	Semi-automated	3
Publication start year	Journal website	NLM Catalog, Ulrichsweb, OCLC WorldCat	Semi-automated	3*
Publication frequency	NLM Catalog	Journal/publisher website	Semi-automated	3*
Link to author instructions	Journal/publisher website	Not applicable	Publisher-level standardization and automation	4
Link to journal home page	Journal/publisher website	Not applicable	Publisher-level standardization and automation	4
Metrics & Indexing				
Impact metrics listed on journal website	Journal/publisher website	Not applicable	Publisher-level standardization and automation	4

Supplemental content to
J Med Libr Assoc. Apr;108(2):dx.doi.org/10.5195/jmla.2020.815
www.jmla.mlanet.org
© Koonce, Blasingame, Zhao, Williams, Su, DesAutels, Giuse, Clark, Fox, Giuse 2020

Field name	Primary source	Additional/confirmatory sources	Level of automation	Stage of automation implementation
Verifiable JCR impact factor	Clarivate JCR impact factor database	Not applicable	Semi-automated	3
MEDLINE indexing status	NLM Catalog	Not applicable	Automated	2
Indexed in SCIE	Clarivate's SCIE journal list	Not applicable	Semi-automated	3
Publication Policies				
Statement of peer-review policy	Journal website	OAPSA website	Publisher-level standardization and automation	4
Adheres to ICMJE recommendations	ICMJE list of journals stating that they follow the ICMJE recommendations	Journal/publisher website	Publisher-level standardization and automation/manual	4
COPE member	COPE website	Not applicable	Semi-automated	3
Archived in PubMed Central	PubMed Central Journals list	Not applicable	Automated	2
Participates in national and international archiving services (e.g., CLOCKSS)	The Keepers Registry	Not applicable	Automated	2
Open Access				
Open access publication	Journal/publisher website	Not applicable	Publisher-level standardization and automation	4
OASPA member	OASPA website	Not applicable	Publisher-level standardization and automation	4

JMLA Journal of the Medical Library Association

Supplemental content to
J Med Libr Assoc. Apr;108(2):dx.doi.org/10.5195/jmla.2020.815
www.jmla.mlanet.org
© Koonce, Blasingame, Zhao, Williams, Su, DesAutels, Giuse, Clark, Fox, Giuse 2020

Field name	Primary source	Additional/confirmatory sources	Level of automation	Stage of automation implementation
Included in DOAJ	DOAJ website	Not applicable	Automated	2
DOAJ seal	DOAJ website	Not applicable	Automated	2
Clearly describes APCs	Journal/publisher website	Not applicable	Publisher-level standardization and automation	4
Statement of article copyright holder	Journal/publisher website	Not applicable	Publisher-level standardization and automation	4
Creative Commons license(s) offered	Journal/publisher website	Not applicable	Publisher-level standardization and automation	4

Abbreviations: APCs=article processing charges, COPE=Committee on Publication Ethics, DOAJ=Directory of Open Access Journals, ICMJE=International Committee of Medical Journal Editors, ISSN=International Standard Serial Number, JCR=Journal Citation Reports, NLM=National Library of Medicine, OCLC=Online Computer Library Center, OASPA=Open Access Scholarly Publishing Association, SCIE=Science Citation Index Expanded.

* Imported data for corporate author, publication start year, and publication frequency were verified in stage 4.

JMLA Journal of the Medical Library Association

SC

Supplemental content to
J Med Libr Assoc. Apr;108(2):dx.doi.org/10.5195/jmla.2020.815
www.jmla.mlanet.org
© Koonce, Blasingame, Zhao, Williams, Su, DesAutels, Giuse, Clark, Fox, Giuse 2020

Supplemental Appendix A References

1. Laine C, Winker MA. Identifying predatory or pseudo-journals. Biochem Med (Zagreb). 2017 Jun 15;27(2):285–91. DOI: http://dx.doi.org/10.11613/BM.2017.031.
2. Committee on Publication Ethics (COPE) Council, Directory of Open Access Journals (DOAJ), Open Access Scholarly Publishers Association (OASPA), World Association of Medical Editors (WAME). Principles of transparency and best practice in scholarly publishing, version 3 [Internet]. Eastleigh, UK: Committee on Publication Ethics; 2019 Jan [cited 17 Oct 2019]. <https://publicationethics.org/resources/guidelines-new/principles-transparency-and-best-practice-scholarly-publishing>.
3. Shamseer L, Moher D, Maduekwe O, Turner L, Barbour V, Burch R, Clark J, Galipeau J, Roberts J, Shea BJ. Potential predatory and legitimate biomedical journals: can you tell the difference? a cross-sectional comparison. BMC Med. 2017 Mar 16;15(1):28. DOI: http://dx.doi.org/10.1186/s12916-017-0785-9.
4. Think. Check. Submit. [Internet]. Think. Check. Submit.; 2019 [cited 17 Oct 2019]. <https://thinkchecksubmit.org/>.
5. Beaubien S, Eckard M. Addressing faculty publishing concerns with open access journal quality indicators. J Librariansh Sch Commun. 2014 Apr 10;2(2):eP1133. DOI: http://dx.doi.org/10.7710/2162-3309.1133.
6. Open Access Scholarly Publishers Association (OASPA). Membership criteria [Internet]. The Hague, Netherlands: The Association [rev. 7 Aug 2019; cited 17 Oct 2019]. <https://oaspa.org/membership/membership-criteria/>.
7. Shen C, Björk BC. 'Predatory' open access: a longitudinal study of article volumes and market characteristics. BMC Med. 2015 Oct 1;13:230. DOI: http://dx.doi.org/10.1186/s12916-015-0469-2.
8. National Institutes of Health. NIH public access policy details [Internet]. Bethesda, MD: The Institutes [rev. 25 Mar 2016; cited 17 Oct 2019]. <https://publicaccess.nih.gov/policy.htm>.
9. Williamson PO, Minter CIJ. Exploring PubMed as a reliable resource for scholarly communications services. J Med Libr Assoc. 2019 Jan;107(1):16–29. DOI: http://dx.doi.org/10.5195/jmla.2019.433.
10. Directory of Open Access Journals (DOAJ). Information for publishers [Internet]. The Directory [cited 17 Oct 2019]. <https://doaj.org>.
11. International Committee of Medical Journal Editors (ICMJE). Recommendations for the conduct, reporting, editing and publication of scholarly work in medical journals [Internet]. The Committee; 29 Nov 2018 [rev. Dec 2018; cited 17 Oct 2019]. <http://www.icmje.org/recommendations/>.
12. Committee on Publication Ethics (COPE). Core practices [Internet]. Eastleigh, UK: The Committee [cited 17 Oct 2019]. <https://publicationethics.org/core-practices>.
13. CLOCKSS [Internet]. South Orange, NJ: CLOCKSS; 2019 [cited 17 Oct 2019]. <https://clockss.org>.
14. The Keepers Registry [Internet]. Edinburgh, UK: EDINA, ISSN International Centre [updated 10 Jul 2019; cited 17 Oct 2019]. <https://thekeepers.org/>.
15. Directory of Open Access Journals. DOAJ seal is now live on the site [Internet]. The Directory; 6 Nov 2015 [cited 17 Oct 2019]. <https://blog.doaj.org/2015/06/11/doaj-seal-is-now-live-on-the-site/>.
