# Supplementary material for: SPI-Hub™: a gateway to scholarly publishing information
Source: J Med Libr Assoc. 2020 Apr 1;108(2):286–94. doi: 10.5195/jmla.2020.815 (PMC7069808; doi:10.5195/jmla.2020.815)
Supplement: Appendix B [file jmla-108-286-s002.pdf]

Journal of the Medical Library Association

Supplemental content to
J Med Libr Assoc. Apr;108(2):dx.doi.org/10.5195/jmla.2020.815
www.jmla.mlanet.org
© Koonce, Blasingame, Zhao, Williams, Su, DesAutels, Giuse, Clark, Fox, Giuse 2020

# SPI-Hub™: a gateway to scholarly publishing information

**Taneya Y. Koonce, MSLS, MPH; Mallory N. Blasingame, MA, MSIS; Jerry Zhao, MS, MLIS; Annette M. Williams, MLS; Jing Su, MD, MS; Spencer J. DesAutels, MLIS; Dario A. Giuse, Dr.Ing., MS, FACMI; John D. Clark, MS; Zachary E. Fox, MSIS; Nunzia Bettinsoli Giuse, MD, MLS, FACMI, FMLA**

## APPENDIX B

### SPI-Hub™ Frequently Asked Questions (FAQ) feature

SPI-Hub™'s Frequently Asked Questions feature provides answers to questions received from users' feedback that we are working to address in the next version of SPI-Hub.

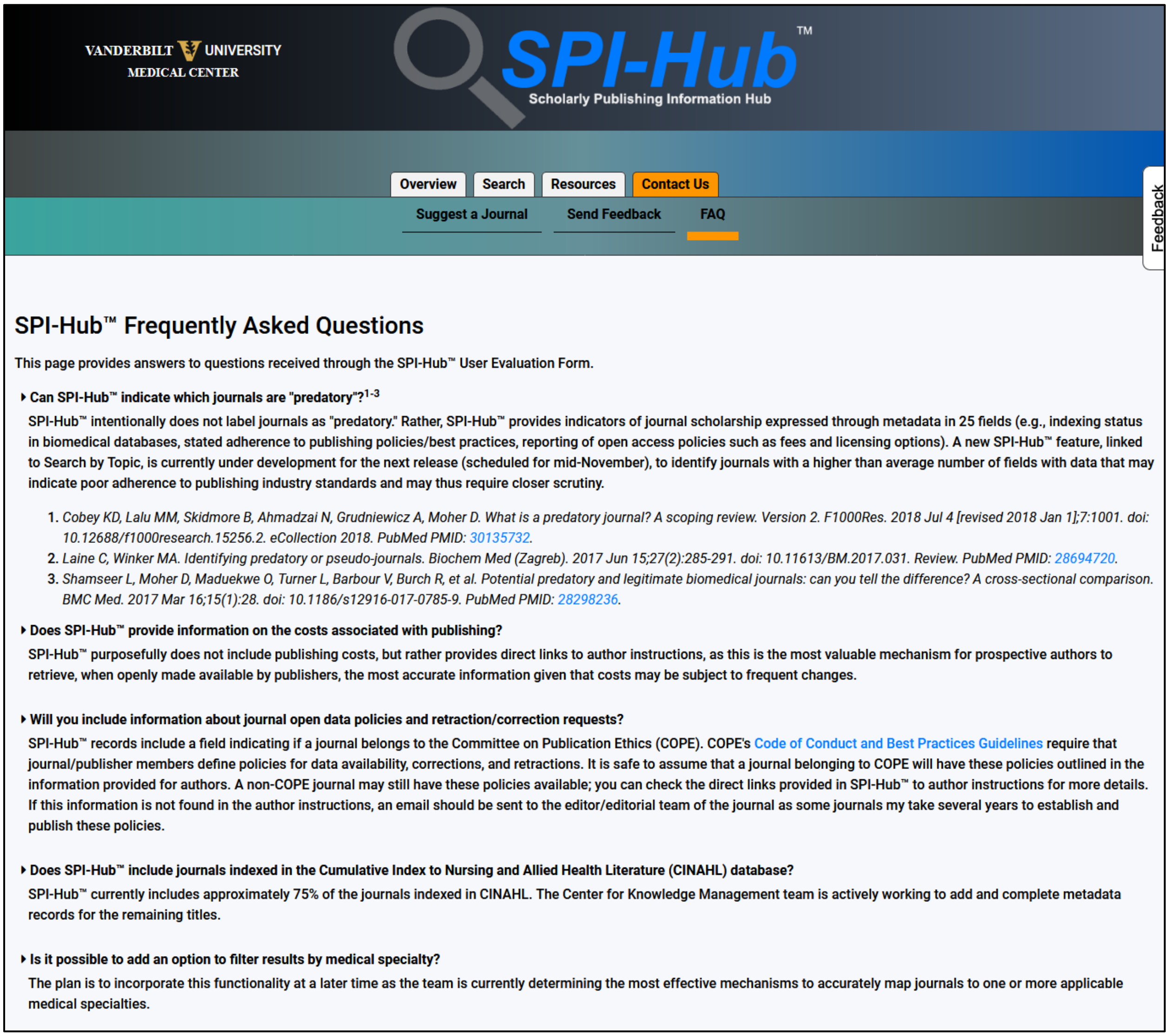



SPI-Hub™ Frequently Asked Questions

This page provides answers to questions received through the SPI-Hub™ User Evaluation Form.

**Can SPI-Hub™ indicate which journals are "predatory"?[1-3]**

SPI-Hub™ intentionally does not label journals as "predatory." Rather, SPI-Hub™ provides indicators of journal scholarship expressed through metadata in 25 fields (e.g., indexing status in biomedical databases, stated adherence to publishing policies/best practices, reporting of open access policies such as fees and licensing options). A new SPI-Hub™ feature, linked to Search by Topic, is currently under development for the next release (scheduled for mid-November), to identify journals with a higher than average number of fields with data that may indicate poor adherence to publishing industry standards and may thus require closer scrutiny.

1. *Cobey KD, Lalu MM, Skidmore B, Ahmadzai N, Grudniewicz A, Moher D. What is a predatory journal? A scoping review. Version 2. F1000Res. 2018 Jul 4 [revised 2018 Jan 1];7:1001. doi: 10.12688/f1000research.15256.2. eCollection 2018. PubMed PMID: 30135732.*
2. *Laine C, Winker MA. Identifying predatory or pseudo-journals. Biochem Med (Zagreb). 2017 Jun 15;27(2):285-291. doi: 10.11613/BM.2017.031. Review. PubMed PMID: 28694720.*
3. *Shamseer L, Moher D, Maduekwe O, Turner L, Barbour V, Burch R, et al. Potential predatory and legitimate biomedical journals: can you tell the difference? A cross-sectional comparison. BMC Med. 2017 Mar 16;15(1):28. doi: 10.1186/s12916-017-0785-9. PubMed PMID: 28298236.*

**Does SPI-Hub™ provide information on the costs associated with publishing?**

SPI-Hub™ purposefully does not include publishing costs, but rather provides direct links to author instructions, as this is the most valuable mechanism for prospective authors to retrieve, when openly made available by publishers, the most accurate information given that costs may be subject to frequent changes.

**Will you include information about journal open data policies and retraction/correction requests?**

SPI-Hub™ records include a field indicating if a journal belongs to the Committee on Publication Ethics (COPE). COPE's Code of Conduct and Best Practices Guidelines require that journal/publisher members define policies for data availability, corrections, and retractions. It is safe to assume that a journal belonging to COPE will have these policies outlined in the information provided for authors. A non-COPE journal may still have these policies available; you can check the direct links provided in SPI-Hub™ to author instructions for more details. If this information is not found in the author instructions, an email should be sent to the editor/editorial team of the journal as some journals my take several years to establish and publish these policies.

**Does SPI-Hub™ include journals indexed in the Cumulative Index to Nursing and Allied Health Literature (CINAHL) database?**

SPI-Hub™ currently includes approximately 75% of the journals indexed in CINAHL. The Center for Knowledge Management team is actively working to add and complete metadata records for the remaining titles.

**Is it possible to add an option to filter results by medical specialty?**

The plan is to incorporate this functionality at a later time as the team is currently determining the most effective mechanisms to accurately map journals to one or more applicable medical specialties.
