# Supplementary material for: SPI-Hub™: a gateway to scholarly publishing information
Source: J Med Libr Assoc. 2020 Apr 1;108(2):286–94. doi: 10.5195/jmla.2020.815 (PMC7069808; doi:10.5195/jmla.2020.815)
Supplement: Appendix C [file jmla-108-286-s003.pdf]

JMLA Journal of the Medical Library Association

Supplemental content to
J Med Libr Assoc. Apr;108(2):dx.doi.org/10.5195/jmla.2020.815
www.jmla.mlanet.org
© Koonce, Blasingame, Zhao, Williams, Su, DesAutels, Giuse, Clark, Fox, Giuse 2020

SPI-Hub™: a gateway to scholarly publishing information

Taneya Y. Koonce, MSLS, MPH; Mallory N. Blasingame, MA, MSIS; Jerry Zhao, MS, MLIS; Annette M. Williams, MLS; Jing Su, MD, MS; Spencer J. DesAutels, MLIS; Dario A. Giuse, Dr.Ing., MS, FACMI; John D. Clark, MS; Zachary E. Fox, MSIS; Nunzia Bettinsoli Giuse, MD, MLS, FACMI, FMLA

APPENDIX C

Sample Knowledge Management Journal Record™

VANDERBILT UNIVERSITY MEDICAL CENTER

SPI-Hub™
Scholarly Publishing Information Hub

Back | Home | **General Information** | Metrics & Indexing | Publication Policies | Open Access

Journal title:	Journal of the American Medical Informatics Association
Corporate author(s):	American Medical Informatics Association (AMIA)
ISSN:	1067-5027(Print); 1527-974X(Electronic)
Scope/Aims URL:	Journal scope/aims link
Publisher:	Oxford University Press
Publication start year:	1994
Publication frequency:	Monthly
Link to author instructions:	Author instructions link
Link to journal homepage:	Journal homepage link

Back | Home | General Information | **Metrics & Indexing** | Publication Policies | Open Access

Journal title:	Journal of the American Medical Informatics Association
Impact metrics listed on website:	Journals from this publisher generally report one or both of the following metrics: Journal Citation Reports (JCR) 2-Year Journal Impact Factor or Journal Citation Reports (JCR) 5-Year Journal Impact Factor. See journal website for details.
Verifiable impact factor from Journal Citation Reports:	Yes
MEDLINE indexing status:	Currently indexed for MEDLINE.
Indexed in Science Citation Index Expanded:	Yes

Back | Home | General Information | Metrics & Indexing | **Publication Policies** | Open Access

Journal title:	Journal of the American Medical Informatics Association
Statement of peer review policy:	Yes
Adheres to ICMJE recommendations:	Yes
Committee on Publication Ethics (COPE) journal member:	Yes
Archived in PubMed Central:	Archives NIH-funded articles only
Participates in national and international archiving services (e.g., CLOCKSS):	Yes (view details)

Back | Home | General Information | Metrics & Indexing | Publication Policies | **Open Access**

Journal title:	Journal of the American Medical Informatics Association
Open access publication:	Paid open access available
Open Access Scholarly Publishing Association (OASPA) member:	Yes
Included in the Directory of Open Access Journals (DOAJ):	Not applicable
Directory of Open Access Journals seal:	Not applicable
Clearly describes Article Processing Charges (APCs):	Yes
Statement of article copyright holder:	Yes
Creative Commons license(s) offered:	Multiple choices offered. See journal website for details.

Record last updated: October 04, 2019
