# Supplementary material for: SPI-Hub™: a gateway to scholarly publishing information
Source: J Med Libr Assoc. 2020 Apr 1;108(2):286–94. doi: 10.5195/jmla.2020.815 (PMC7069808; doi:10.5195/jmla.2020.815)

JMLA Journal of the Medical Library Association

SC
Supplemental content to
J Med Libr Assoc. Apr;108(2):dx.doi.org/10.5195/jmla.2020.815
www.jmla.mlanet.org
© Koonce, Blasingame, Zhao, Williams, Su, DesAutels, Giuse, Clark, Fox, Giuse 2020

SPI-Hub™: a gateway to scholarly publishing information

Taneya Y. Koonce, MSLS, MPH; Mallory N. Blasingame, MA, MSIS; Jerry Zhao, MS, MLIS; Annette M. Williams, MLS; Jing Su, MD, MS; Spencer J. DesAutels, MLIS; Dario A. Giuse, Dr.Ing., MS, FACMI; John D. Clark, MS; Zachary E. Fox, MSIS; Nunzia Bettinsoli Giuse, MD, MLS, FACMI, FMLA

APPENDIX D

Search by Author functionality

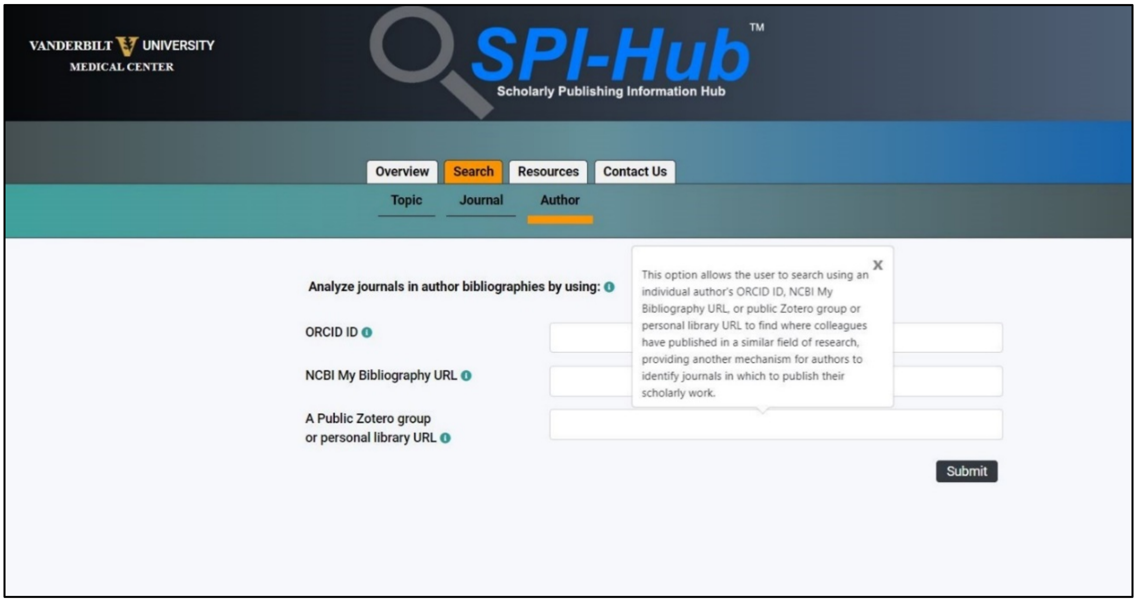

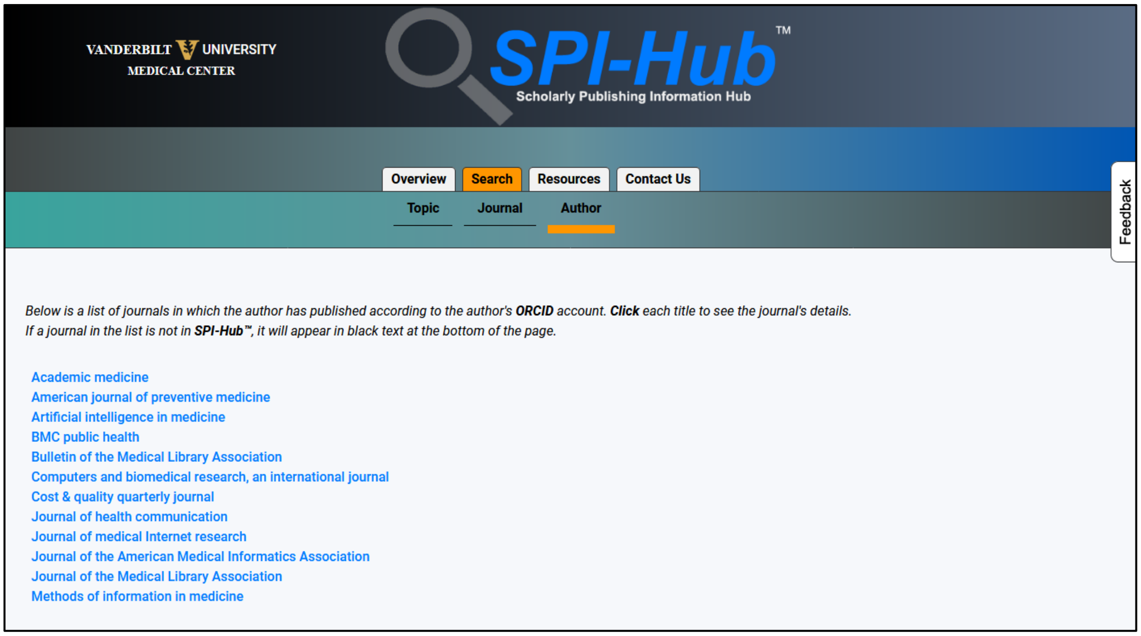

Supplement: Appendix D [file jmla-108-286-s004.pdf]
