# Supplementary material for: SPI-Hub™: a gateway to scholarly publishing information
Source: J Med Libr Assoc. 2020 Apr 1;108(2):286–94. doi: 10.5195/jmla.2020.815 (PMC7069808; doi:10.5195/jmla.2020.815)
Supplement: Appendix E [file jmla-108-286-s005.pdf]

JMLA Journal of the Medical Library Association

SC
Supplemental content to
J Med Libr Assoc. Apr;108(2):dx.doi.org/10.5195/jmla.2020.815
www.jmla.mlanet.org
© Koonce, Blasingame, Zhao, Williams, Su, DesAutels, Giuse, Clark, Fox, Giuse 2020

SPI-Hub™: a gateway to scholarly publishing information

Taneya Y. Koonce, MSLS, MPH; Mallory N. Blasingame, MA, MSIS; Jerry Zhao, MS, MLIS; Annette M. Williams, MLS; Jing Su, MD, MS; Spencer J. DesAutels, MLIS; Dario A. Giuse, Dr.Ing., MS, FACMI; John D. Clark, MS; Zachary E. Fox, MSIS; Nunzia Bettinsoli Giuse, MD, MLS, FACMI, FMLA

APPENDIX E

Automation impact study

Methods

The automation impact study was conducted to document the resources needed to complete and maintain SPI-Hub™ records in each of four stages of automation. One study team member (Fox) selected a subset of SPI-Hub records from publishers with at least ten journals, using a random number generator [1], and prepared standardized data completion forms. In order to avoid confounding from variations in how publishers present information on their websites, the publishers were held constant across the randomization process. Records used in stages two to four were from the same publishers identified in stage one. Five study team members (Koonce, Blasingame, Williams, Su, DesAutels) were assigned to complete the same set of records randomly selected for each stage of implementation. Each individual had approximately six months of experience populating SPI-Hub records since the project's inception and was well versed with publishing industry standards and practices.

In the first stage of automation, information for all metadata fields was gathered manually and no data were pre-populated. The journal title field was provided for all records, leaving twenty-four fields for data collection. Records in the second stage of automation had five fields completed, using data from sources where extraction is fully automated (each field's level of automation is described in supplemental Appendix A), leaving nineteen fields for manual collection. In the third stage, an additional eight fields were pre-populated, reducing the number of fields for manual review to eleven. These eight fields are completed using semi-automated methods: data are gathered and uploaded from multiple sources but require additional review to reconcile duplicates, matches, and non-matches.

In stage four, the last eleven fields were semi-automatically populated based on publisher-level policies and practices. In this stage, we evaluated the time required to manually review and complete three fields with data imported from the National Library of Medicine (NLM) Catalog. While most of the data that we have collected from the NLM Catalog are correct, we have noticed some instances where the data for certain fields (i.e., corporate authors, publication start year, and publication frequency) are no longer accurate, as changes have been made at the journal or publisher level and are not yet reflected in the NLM Catalog record, thus requiring us to handle those fields manually. This final stage is reflective of the work flow and maintenance process that we are using for journals issued by large publishers, representing around half of the SPI-Hub database, in which most of the metadata are completed through automated and semi-automated processes, and the few remaining fields are manually validated.

The five study team members each completed the metadata for all records, capturing the time in seconds required to populate each field. The average time spent completing the manually populated portion of the records for each stage was determined for both the individual team members and the group as a whole. Descriptive comparisons were made across stages of implementation and among team members at each stage to evaluate whether any observed reductions in time effort were proportional, regardless of individual differences.

Journal of the Medical Library Association

Supplemental content to
J Med Libr Assoc. Apr;108(2):dx.doi.org/10.5195/jmla.2020.815
www.jmla.mlanet.org
© Koonce, Blasingame, Zhao, Williams, Su, DesAutels, Giuse, Clark, Fox, Giuse 2020

Results

Records for 40 journals were completed by each team member (10 records for each of the 4 stages of automation). On average, 12.16 minutes per journal were required for data gathering in stage 1, which was reduced in each stage, with the time reduced in stage 4 to an average of 1.62 minutes per journal (supplemental Figure 1). The time reduction was also observed for each team member individually (supplemental Figure 2).

Supplemental Figure 1 Average record completion time by automation stage (n=5)

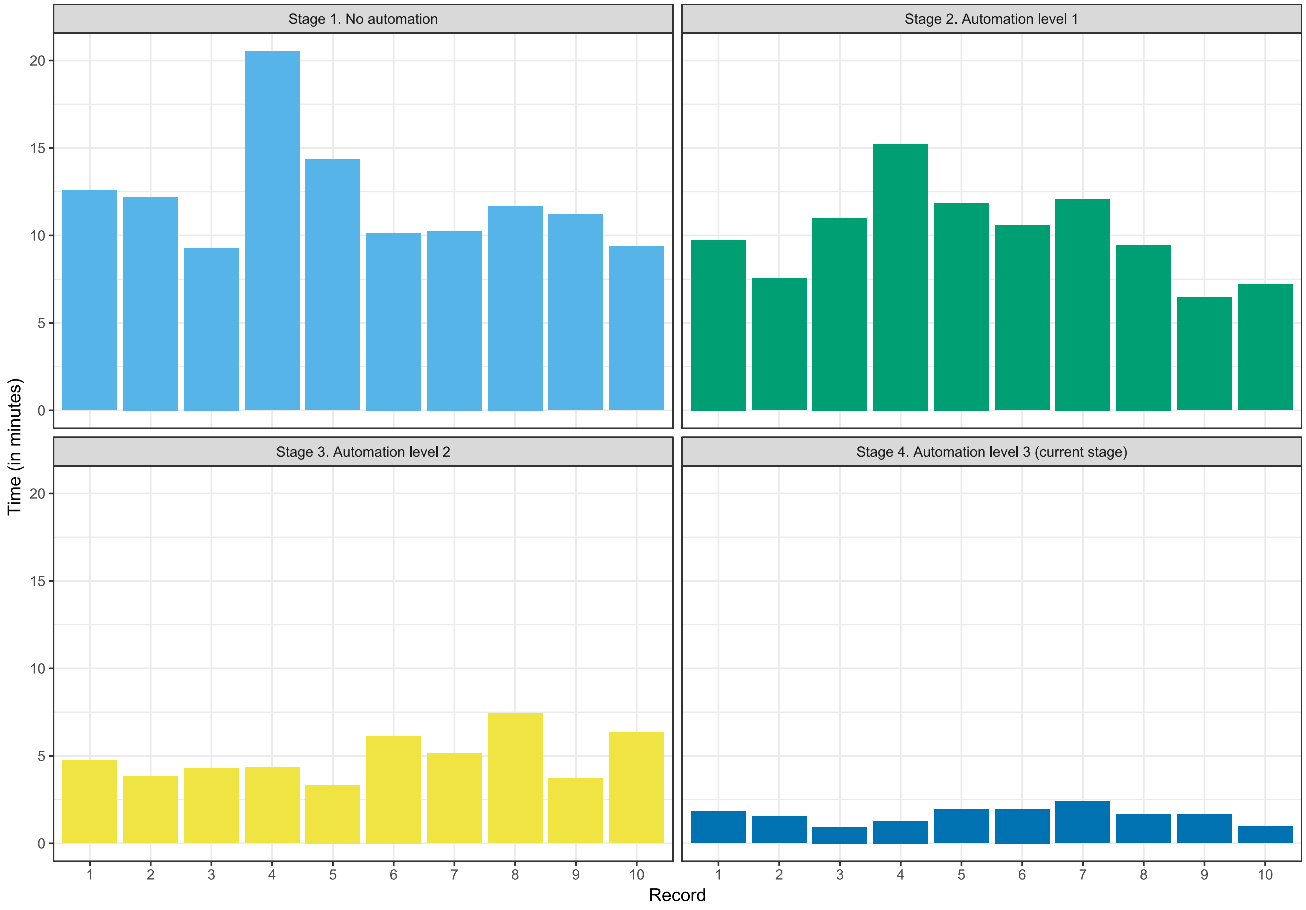

Journal of the Medical Library Association

Supplemental content to
J Med Libr Assoc. Apr;108(2):dx.doi.org/10.5195/jmla.2020.815
www.jmla.mlanet.org
© Koonce, Blasingame, Zhao, Williams, Su, DesAutels, Giuse, Clark, Fox, Giuse 2020

Supplemental Figure 2 Average record completion time by participant

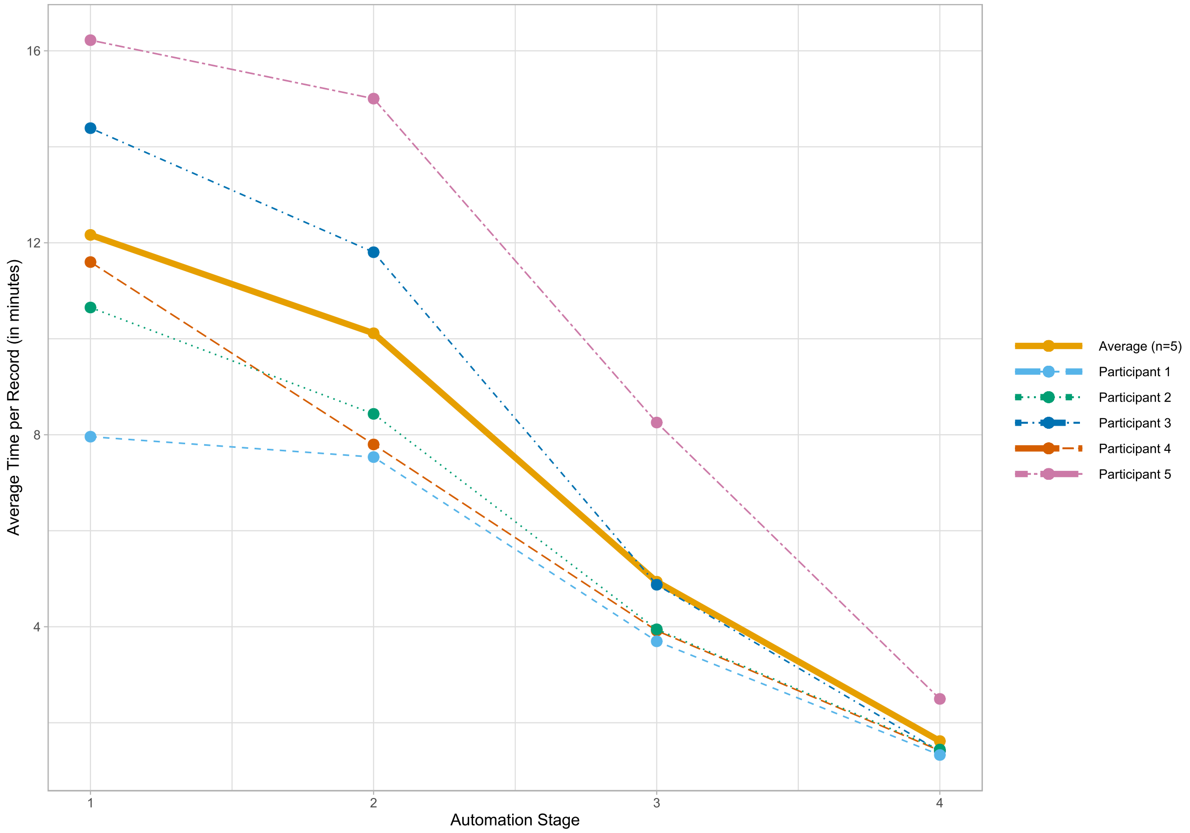

For the three remaining fields requiring manual review after the automation process used for large publishers, we have established a systematic web-based "assembly line" process as the last stage of information collection: a team member is dedicated to one "station" (or field) in the assembly line, and, once completed, the record is automatically moved to the next station for verification and completion via the web-based data acquisition editor. Once a journal record has passed through all three stations, it is fully complete and ready for inclusion in SPI-Hub. To further leverage work performed on the assembly line, knowledge of where station-level data reside on each journal website is used to inform the future conversion of this manual process into automated data capture and verification.

Supplemental Appendix E Reference

1. Randomness and Integrity Services. Random.org [Internet]. Dublin, Ireland: Random.org; 1998– [cited 17 Oct 2019]. <https://www.random.org/>.
