# Supplementary material for: SPI-Hub™: a gateway to scholarly publishing information
Source: J Med Libr Assoc. 2020 Apr 1;108(2):286–94. doi: 10.5195/jmla.2020.815 (PMC7069808; doi:10.5195/jmla.2020.815)
Supplement: Appendix F [file jmla-108-286-s006.pdf]

JMLA Journal of the Medical Library Association

SC
Supplemental content to
J Med Libr Assoc. Apr;108(2):dx.doi.org/10.5195/jmla.2020.815
www.jmla.mlanet.org
© Koonce, Blasingame, Zhao, Williams, Su, DesAutels, Giuse, Clark, Fox, Giuse 2020

SPI-Hub™: a gateway to scholarly publishing information

Taneya Y. Koonce, MSLS, MPH; Mallory N. Blasingame, MA, MSIS; Jerry Zhao, MS, MLIS; Annette M. Williams, MLS; Jing Su, MD, MS; Spencer J. DesAutels, MLIS; Dario A. Giuse, Dr.Ing., MS, FACMI; John D. Clark, MS; Zachary E. Fox, MSIS; Nunzia Bettinsoli Giuse, MD, MLS, FACMI, FMLA

APPENDIX F

SPI-Hub™ user evaluation

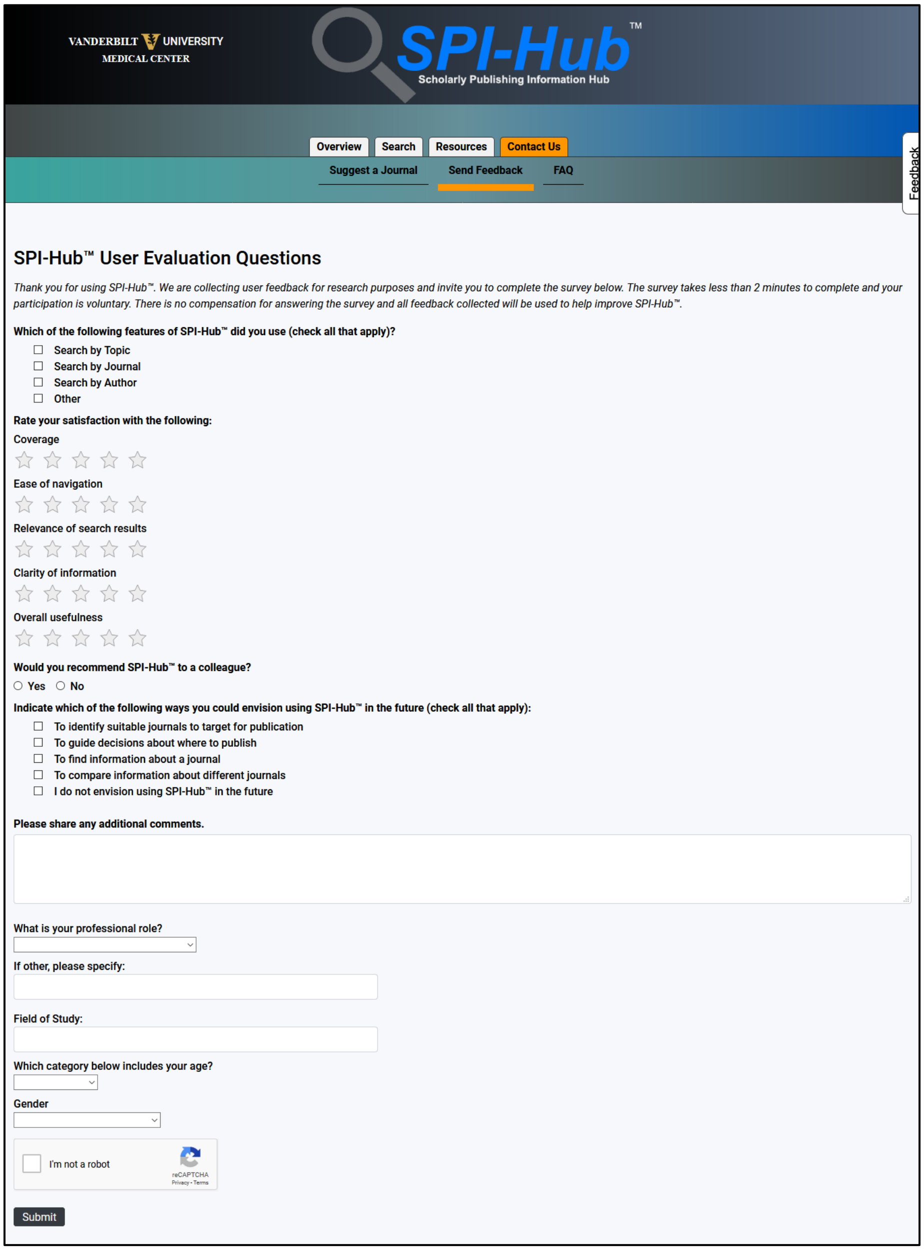
VANDERBILT UNIVERSITY MEDICAL CENTER
SPI-Hub™
Scholarly Publishing Information Hub

Overview | Search | Resources | Contact Us
Suggest a Journal | Send Feedback | FAQ
Feedback

SPI-Hub™ User Evaluation Questions

Thank you for using SPI-Hub™. We are collecting user feedback for research purposes and invite you to complete the survey below. The survey takes less than 2 minutes to complete and your participation is voluntary. There is no compensation for answering the survey and all feedback collected will be used to help improve SPI-Hub™.

Which of the following features of SPI-Hub™ did you use (check all that apply)?

- ☐ Search by Topic
- ☐ Search by Journal
- ☐ Search by Author
- ☐ Other

Rate your satisfaction with the following:

Coverage ☆☆☆☆☆

Ease of navigation ☆☆☆☆☆

Relevance of search results ☆☆☆☆☆

Clarity of information ☆☆☆☆☆

Overall usefulness ☆☆☆☆☆

Would you recommend SPI-Hub™ to a colleague?

○ Yes ○ No

Indicate which of the following ways you could envision using SPI-Hub™ in the future (check all that apply):

- ☐ To identify suitable journals to target for publication
- ☐ To guide decisions about where to publish
- ☐ To find information about a journal
- ☐ To compare information about different journals
- ☐ I do not envision using SPI-Hub™ in the future

Please share any additional comments.

What is your professional role?

If other, please specify:

Field of Study:

Which category below includes your age?

Gender

I'm not a robot
reCAPTCHA
Privacy - Terms

Submit
